# Rise and dissemination of aminoglycoside resistance: the *aac(6′)-Ib* paradigm

**DOI:** 10.3389/fmicb.2013.00121

**Published:** 2013-05-17

**Authors:** María S. Ramirez, Nikolas Nikolaidis, Marcelo E. Tolmasky

**Affiliations:** Department of Biological Science, Center for Applied Biotechnology Studies, College of Natural Sciences and Mathematics, California State University FullertonFullerton, CA, USA

**Keywords:** antibiotic resistance, aminoglycoside, inhibition, acetyltransferase, mobile elements, integron, transposon

## Abstract

Enzymatic modification is a prevalent mechanism by which bacteria defeat the action of antibiotics. Aminoglycosides are often inactivated by aminoglycoside modifying enzymes encoded by genes present in the chromosome, plasmids, and other genetic elements. The AAC(6′)-Ib (aminoglycoside 6′-*N*-acetyltransferase type Ib) is an enzyme of clinical importance found in a wide variety of gram-negative pathogens. The AAC(6′)-Ib enzyme is of interest not only because of his ubiquity but also because of other characteristics, it presents significant microheterogeneity at the N-termini and the *aac(6′)-Ib* gene is often present in integrons, transposons, plasmids, genomic islands, and other genetic structures. Excluding the highly heterogeneous N-termini, there are 45 non-identical AAC(6′)-Ib related entries in the NCBI database, 32 of which have identical name in spite of not having identical amino acid sequence. While some variants conserved similar properties, others show dramatic differences in specificity, including the case of AAC(6′)-Ib-cr that mediates acetylation of ciprofloxacin representing a rare case where a resistance enzyme acquires the ability to utilize an antibiotic of a different class as substrate. Efforts to utilize antisense technologies to turn off expression of the gene or to identify enzymatic inhibitors to induce phenotypic conversion to susceptibility are under way.

## Aminoglycosides and resistance

Aminoglycosides are bactericidal antibiotics that affect translation fidelity and, according to recent data, they may also stimulate the production of highly deleterious hydroxyl radicals (Vakulenko and Mobashery, 2003; Magnet and Blanchard, 2005; Jana and Deb, 2006; Kohanski et al., 2007; Majumder et al., 2007). Aminoglycosides are used to treat infections caused by gram-negative bacilli and, in combination with β-lactams or vancomycin, to treat some gram-positive pathogens, mainly staphylococci (Yao and Moellering, 2007). Since a step in the uptake process requires functional respiration, the spectrum of action of aminoglycosides is limited to aerobic bacteria (Muir et al., 1984). In addition to their most common uses, aminoglycosides can be utilized to treat diseases such as tuberculosis (Menzies et al., 2009; Brossier et al., 2010), plague, tularemia, brucellosis, endocarditis caused by enterococci, and others (Vakulenko and Mobashery, 2003; Yao and Moellering, 2007; Ramirez and Tolmasky, 2010). The fact that aminoglycosides also cause a decrease in eukaryotic translational fidelity permitted to initiate efforts to developed them as drugs to treat nonsense mutation related genetic disorders such as cystic fibrosis and Duchenne muscular dystrophy (Rich et al., 1990; Kellermayer, 2006; Hermann, 2007; Kondo et al., 2007; Zingman et al., 2007; Bidou et al., 2012; Kandasamy et al., 2012). A chemical labyrinthectomy using intratympanic injection of aminoglycosides is used when most treatments of Ménière's disease fail (Huon et al., 2012; Pacheu-Grau et al., 2012). Aminoglycoside-based drugs are also inhibitors of reproduction of the HIV virus, a property that could result in their utilization in the treatment of AIDS patients (Houghton et al., 2010).

The basic chemical structure of aminoglycosides is characterized by the presence of an aminocyclitol nucleus (streptamine, 2-deoxystreptamine, or streptidine) linked to amino sugars through glycosidic bonds. However, other compounds with different basic structures are also included within the aminoglycosides family, e.g., spectinomycin, an aminocyclitol not linked to amino sugars or compounds containing the aminocyclitol fortamine (Bryskier, 2005). They reach the cytoplasm of the bacterial cell in a three-step process, of which the first one is energy-independent and the following two are energy-dependent (Taber et al., 1987; Vakulenko and Mobashery, 2003; Ramirez and Tolmasky, 2010). At the molecular level, the action of aminoglycosides is characterized by interactions between the antibiotic molecule and the 16S rRNA. Although for all aminoglycosides the effect of this interaction is a change of conformation of the decoding A site producing one that resembles the closed state induced by interaction between cognate tRNA and mRNA, it must be noted that not all aminoglycosides seem to bind the same sites of the 16S rRNA. The consequence of the conformational change induced by the interaction 16S rRNA-aminoglycoside is the reduction of the proofreading capabilities of the ribosome, which in turns results in high levels of mistranslation (Bakker, 1992; Busse et al., 1992; Vakulenko and Mobashery, 2003; Vicens and Westhof, 2003; Magnet and Blanchard, 2005; Majumder et al., 2007; Zaher and Green, 2009; Ramirez and Tolmasky, 2010). Other molecular effects of some aminoglycosides have been described but it is not clear if some of them are not secondary to protein mistranslation. They include inhibition of 30S ribosomal subunit assembly, induction of RNA cleavage, or interference with the action of RNase P (Mikkelsen et al., 1999; Mehta and Champney, 2003; Belousoff et al., 2009).

Aminoglycosides are powerful tools against infections (Labaune et al., 2001; Avent et al., 2011) but unfortunately the levels of resistance are growing and in consequence failure of treatments with aminoglycosides is becoming more common (Galani et al., 2002; van ‘t Veen et al., 2005; Tolmasky, 2007a; Soler Bistue et al., 2008). Bacteria have developed numerous mechanisms to resist the action of aminoglycosides and cells can possess the genetic determinants for several of them enhancing the levels of resistance and making it very difficult to overcome all of them. Enzymatic inactivation by acetylation, adenylylation, or phosphorylation at different locations of the aminoglycoside molecule is among the most clinically relevant strategies bacteria use to resist the action of these antibiotics (Shaw et al., 1993; Vakulenko and Mobashery, 2003; Tolmasky, 2007a; Ramirez and Tolmasky, 2010; Chen et al., 2011; Chiang et al., 2013). The enzymes that catalyze these reactions are collectively known as aminoglycoside modifying enzymes. Other well studied mechanisms are: (1) mutation of the 16S rRNA or ribosomal proteins modify the target eliminating or reducing the interaction with the antibiotic molecule (O'Connor et al., 1991); (2) methylation of 16S rRNA, a mechanism found in most aminoglycoside-producing organisms and in clinical strains (Schmitt et al., 2009; Wachino and Arakawa, 2012); (3) reduced permeability to the antibiotic molecule by modification of the permeability of the outer membrane or diminished inner membrane transport (Hancock, 1981; Taber et al., 1987; Macleod et al., 2000; Over et al., 2001); (4) export outside the cell by active efflux pumps (Hocquet et al., 2003; Morita et al., 2012; Wachino and Arakawa, 2012); (5) sequestration by tight binding to a low active aminoglycoside acetyltransferase (Magnet et al., 2003); and (6) extracellular DNA shielding in biofilms (Chiang et al., 2013).

The general characteristics of all known aminoglycoside modifying enzymes have been recently reviewed (Ramirez and Tolmasky, 2010). This review will focus on the aminoglycoside 6′-*N*-acetyltransferase type Ib [AAC(6′)-Ib], which is of great clinical relevance and it is found in over 70% of AAC(6′)-I-producing gram-negative clinical isolates (Vakulenko and Mobashery, 2003), and has been the subject of numerous studies (Tolmasky, 2007a; Cambray and Mazel, 2008; Ramirez and Tolmasky, 2010).

## The AAC(6′)-Ib protein

The aminoglycoside *N*-acetyltransferases (AAC) belong to the GCN5-related N-acetyltransferase superfamily, also known as GNAT. This is a large group of enzymes that includes about 10,000 proteins from all kinds of organisms that share the property to catalyze the acetylation of a primary amine in numerous acceptor molecules using acetyl CoA as donor substrate (Neuwald and Landsman, 1997; Dyda et al., 2000; Vetting et al., 2005). The AACs are subdivided in groups based on the position where the acetyl group is transferred in the acceptor aminoglycoside molecule. Known AACs catalyze acetylation at the 1 [AAC(1)], 3 [AAC(3)], 2′ [AAC(2′)], or 6′ [AAC(6′)] positions (Shaw et al., 1993; De Pascale and Wright, 2010; Ramirez and Tolmasky, 2010). AAC(6′) enzymes are the most numerous group of AACs, more than 40 have been described, and can be found in gram-negatives as well as gram-positives (Shaw et al., 1993; Miller et al., 1997; Wright, 1999; Tolmasky, 2007a; Ramirez and Tolmasky, 2010). AAC(6′) enzymes are subdivided in two groups, AAC(6′)-I and AAC(6′)-II, which are differentiated by the profile of the aminoglycosides inactivated. With a few exceptions, AAC(6′)-I enzymes specify resistance to several aminoglycosides plus amikacin and gentamicin C1a and C2 but not to gentamicin C1 (Shaw et al., 1993). On the other hand, AAC(6′)-II enzymes catalyze acetylation of all forms of gentamicin but not of amikacin (Rather et al., 1992). In addition, enzymes with extended spectrum that may merit addition of new subclasses of AAC(6′)-I enzymes have been recently described (Casin et al., 2003; Robicsek et al., 2006; Strahilevitz et al., 2009). Phylogenetic analyses divided the AAC(6′) enzymes into three clades. However, with the information available it is still not clear if all AAC(6′) enzymes evolved from a single origin or the three groups are less related and the 6′ acetylating activity has evolved independently at least three times (Salipante and Hall, 2003). According to the phylogenetic analyses recently communicated by Salipante and Hall (Salipante and Hall, 2003) the AAC(6′)-Ib is most closely related to AAC(6′)-IIa AAC(6′)-IIb, AAC(6′)-IIc, and AAC(6′)-IId.

There are numerous variants of AAC(6′)-Ib, many of them identified by modifications in the name of the enzyme such as the addition of subscripts or a prime symbol superscript (Cambray and Mazel, 2008; Ramirez and Tolmasky, 2010). However, a large number of versions of the protein, or predicted protein, have all been named AAC(6′)-Ib, which can be a source of confusion or indetermination. These variants mainly differ at the N-terminus, however one should be careful when considering these differences because not in all of them the N-terminus has been experimentally determined (Dery et al., 1997; Casin et al., 2003; Soler Bistue et al., 2006; Maurice et al., 2008). Table 1 shows a list of the *aac(6′)-Ib* gene versions found in different genetic environments and bacterial species. Some variants differing at the N-termini such as AAC(6′)-Ib_3_, AAC(6′)-Ib_4_, AAC(6′)-Ib_6_, and AAC(6′)-Ib_7_ have been compared and it was found that they have similar behavior (Casin et al., 1998) but variations as small as one or two amino acids at key positions proved to be of high relevance (Table 2). For example, the AAC(6′)-Ib_11_ found in *S. Typhimurium* has L and S residues at positions 118 and 119 as opposed to Q and L or Q and S, the amino acids present at these positions in all previously described enzymes, acquired an extended resistance spectrum that includes all three gentamicin forms (Casin et al., 2003). (Amino acid numbers throughout the text are based on the sequence corresponding to accession number AF479774.) Another example worth mentioning is the AAC(6′)-Ib', originally found in *Pseudomonas fluorescens* BM2687, but previously generated by site-directed mutagenesis in the laboratory (Table 1). This protein has a L to S substitution at amino acid 119 that confers the enzyme an AAC(6′)-II profile, i.e., the enzyme confers resistance to gentamicin but not amikacin (Rather et al., 1992; Lambert et al., 1994). A highly surprising effect occurred in the natural variant known as AAC(6′)-Ib-cr, which has the modifications W104R and N181Y (Tables 1, 2). The substrate spectrum was expanded to include quinolone antibiotics, crossing the barrier from the aminoglycosides (Robicsek et al., 2006). Since the first detection of the AAC(6′)-Ib-cr variant there have been numerous reports of its presence, and variants of it, across the world in different genetic environments suggesting an extraordinary ability to disseminate (Quiroga et al., 2007; Cattoir and Nordmann, 2009; Strahilevitz et al., 2009; Rodriguez-Martinez et al., 2011; Ruiz et al., 2012; De Toro et al., 2013). Furthermore, there have been cases where a strain was found to simultaneously include genes coding for AAC(6′)-Ib and AAC(6′)-Ib-cr (Kim et al., 2011). AAC(6′)-Ib is also found fused to the C-terminal end of AAC(3)-Ib protein within a class I integron found in a *Pseudomonas aeruginosa* strain (Dubois et al., 2002) and to the C-terminus of the AAC(6′)-30 also within a *P. aeruginosa* class I integron (Mendes et al., 2004).

**Table 1:** **AAC(6′)-Ib variants**.

**Number**	**AAC(6′)-Ib enzyme**	**Gene allele**	**Genetic localization**	**Species**	**Reference**
1	NP_608307	*aac(6′)-Ib*	pJHCMW1::Tn*1331*,	*K. pneumoniae, En.* spp.,	NP_608307,
			pKPN4,	*Pseudomonas putida,*	YP_001338668,
			pMET1::Tn*1331.2*,	*Proteus mirabilis*	YP_001928078,
			pKlebpneu15S,		YP_001928081,
			pR23::Tn*1331*,		YP_002286819,
			pAAC154:: ΔTn*1331*,		YP_004455304,
			pColEST258,		YP_006958960,
			pJHC-MW1,		YP_006959190,
			Tn*1332*,		ZP_14492679 (contig),
			class 1 integron,		ZP_14498301 (contig),
			pRMH712::Tn*1331*,		ZP_14503930 (contig),
			SGI1-V::class 1 integron		ZP_14509538 (contig),
					ZP_14515174 (contig),
					ZP_14520767 (contig),
					ZP_14526392 (contig),
					ZP_14531781 (contig),
					ZP_14537604 (contig),
					ZP_14543183 (contig),
					ZP_14548764 (contig),
					ZP_14554292 (contig),
					ZP_14559831 (contig),
					ZP_14565429 (contig),
					ZP_14571055 (contig),
					ZP_14576504 (contig),
					ZP_14581777 (contig),
					ZP_14587733 (contig),
					ZP_14593028 (contig),
					ZP_14598930 (contig),
					ZP_19010829 (contig),
					AAC6_KLEPN,
					AF479774_5, AAA69747
					AAA98404, ABA54975,
					ABR80438, ACB55476,
					ACB55479, ACI63081,
					ACL36604, ADK35766,
					AED98720, AED99555,
					AEG74535, AEW43367,
					EJJ31842 (contig),
					EJJ31842 (contig),
					EJJ31884 (contig),
					EJJ31888 (contig),
					EJJ48672 (contig),
					EJJ48714 (contig),
					EJJ49532 (contig),
					EJJ65671 (contig),
					EJJ65889 (contig),
					EJJ68295 (contig),
					EJJ79581 (contig),
					EJJ81436 (contig),
					EJJ85464 (contig),
					EJJ96302 (contig),
					EJJ96595 (contig),
					EJK03278 (contig),
					EJK13161 (contig),
					EJK16025 (contig),
					EJK18863 (contig),
					EJK30890 (contig),
					EJK33635 (contig),
					EKV58688 (contig)
2	YP_002286969	*aac(6′)-Ib*	p12::Tn*1331*	*K. pneumoniae, E. coli*	YP_002286969,
					ZP_16459764 (genomic scaffold), ZP_19016755 (contig), ACI63027,
					EGB78408 (contig),
					EKV58524 (contig)
3	AAA26550	*aac(6′)-Ib*	pAZ007	*Serratia marcescens*	AAA26550
4	AAR18814	*aac(6′)-Ib*	pKP31::class 1 integron	*K. pneumoniae*	AAR18814
5	CBI63199	*aac(6′)-Ib*	Class 1 integron	*P. aeruginosa*	CBI63199
6	CBI63201	*aac(6′)-Ib*	Class 1 integron	*P. aeruginosa*	CBI63201
7	CBI63203	*aac(6′)-Ib*	Class 1 integron	*P. aeruginosa*	CBI63203
8	ABG77519	*aac(6′)-Ib*	Class 1 integron	*P. aeruginosa*	ABG77519
9	CBL95252	*aac(6′)-Ib*	Class 1 integron	*P. aeruginosa*	CBL95252
10	CBL95256	*aac(6′)-Ib*	Class 1 integron	*P. aeruginosa*	CBL95256
11	CBI63204	*aac(6′)-Ib*	Class 1 integron	*P. aeruginosa*	CBI63204
12	CBI63202	*aac(6′)-Ib*	Class 1 integron	*P. aeruginosa*	CBI63202
13	YP_003937697	*aac(6′)-Ib*	pETN48:: Δclass 1 integron	*E. coli*	YP_003937697,
					CBX36023
14	ADC80806	*aac(6′)-Ib*	pRYC103T24::class 1 integron In4-like,	*E. coli,* uncultured bacterium	ADC80806, AFR44153
			pKSP212::class 1 integron		
15	YP_005797131	*aac(6′)-Ib*	Class 1 integron (Chromosome)	*A. baumannii*	YP_005797131,
					AEN92376
16	YP_005525242	*aac(6′)-Ib*	Class 1 integron (Chromosome)	*A. baumannii*	YP_005525242,
					YP_006289231,
					YP_006848983,
					ZP_11603605 (contig),
					ZP_16142456 (contig),
					ZP_16146111 (contig),
					EGK45756 (seq0044),
					AEP05746, AFI94936,
					EKE64317 (contig),
					EKE64588 (contig),
					AFU38752
17	NP_863005	*aac(6′)-Ib*	p1658/97::class 1 integron, class 1 integron (Chromosome), class 1 integron, plasmid In238a	*E. coli, A. baumannii,*	NP_863005,
				*K. pneumoniae,*	YP_001844882,
				*K. oxytoca, En. cloacae*	AAO49600, ACZ55927,
					ACZ64698, AFS33307
18	ADC80825	*aac(6′)-Ib*	pRYC103T24::class 1 integron	*E. coli*	ADC80825
19	YP_002791392	*aac(6′)-Ib*	pEC-IMP::class 1 integron,	*En. cloacae, Salmonella enterica subsp. enterica* serovar *Bredeney, P. aeruginosa*	YP_002791392,
			pEC-IMPQ::class 1 integron, pb1004::class 1 integron, class 1 integron		YP_002791702,
					ACF59628, ACO54016,
					ACO54326, ADF47469
20	AEO50496	*aac(6′)-Ib*	Class 1 integron	*Se. marcescens*	AEO50496
21	ACB41759	*aac(6′)-Ib*	Class 1 integron	*E. coli*	ACB41759
22	BAL45797	*aac(6′)-Ib*	pKPI-6::class 1 integron	*K. pneumoniae*	BAL45797
23	AAC46343	*aac(6′)-Ib*	Class 1 integron	*P. aeruginosa*	AAC46343
24	AAD02244	*aac(6′)-Ib9*	Class 1 integron	*P. aeruginosa*	AAD02244
25	YP_003108195	*aac(6′)-Ib-cr*	pEK516, pEK499,	*E. coli, K. pneumoniae*	YP_003108195,
			pEC_L8, pUUH239.2		YP_003108338,
					YP_003829182,
					YP_005351453,
					ACQ41894, ACQ42045,
					ADL14076, AET17280
26	ZP_18354173	*aac(6′)-Ib-cr*		*K. pneumoniae*	ZP_18354173 (genomic scaffold), EKF76226
27	ACD56150	*aac(6′)-Ib-cr*	pHS1387::class 1 integron	*Escherichia coli*	ACD56150
28	ADY02579	*aac(6′)-Ib-cr*	Class 1 integron	*Aeromonas media*	ADY02579
29	NP_957555	*aac(6′)-Ib-cr*	pC15-1a, pKP96::class 1	*Escherichia coli,*	NP_957555,
			integron, pNDM-MAR,	*K. pneumoniae, Kluyvera*	YP_002332851,
			pGUE-NDM, pKDO1,	*ascorbata,* mixed culture	YP_005352168,
			pHe96, pKas96,	bacterium, *K. oxytoca,*	YP_006953881,
			pECZ6-1::class 1	*Se. rubidaea, En. cloacae,*	YP_006973732,
			integron, Class 1	*Aeromonas*	AAR25030, ABC17627,
			integron, pLC108::class 1	*allosaccharophila,*	ABM47029, ABY74389,
			integron, pJIE101,	*Providencia* spp., *Shigella* spp., *En. aerogenes*	ACD03312, ACD03322,
					ACM24788, ACT97328,
					ACT97332, ACT97345,
					ACT97681, ACV60575,
					ADA60222, ADE44336,
					ADP30789, ADU16107,
					ADU16118, ADY02556,
					AEC49701, AEC49704,
					AEL33522, AEO45791,
					AEO79936, AEO79967,
					AEP16466, AER36609,
					AEU10750, AEU10754,
					AFB82784, AFC38861,
					AFI72862, AFV52812,
					AFV70394
30	1V0C_A	*aac(6′)-Ib*		*Escherichia coli* Chain A, Structure	1V0C_A, 2BUE_A,
					2VQY_A
31	YP_006501621		pKOX_R1::class 1 integron, class 1 integorn,	*K. oxytoca,*	YP_006501621,
				*K. pneumoniae*	AFM57748, AFN35014
32	ABC54722	*aac(6′)-Ib*	pAS1::InVC117	*Vibrio cholerae*	ABC54722
33	BAE66666	*aac(6′)-Ib*	Class 1 integron	*Vibrio cholerae* O1	BAE66666
34	YP_007232190	*aac(6′)-Ib*	pPC9	*P. putida*	YP_007232190
35	ZP_16084267	*aac(6′)-Ib*	Class 1 integron (Chromosome)	*A. baumannii*	ZP_16084267 (contig),
					ZP_16086960 (contig),
					ZP_16140385 (contig),
					EKA73751 (contig),
					EKK08901 (contig),
					EKK18976 (contig)
36	AFS51540	*aac(6′)-Ib9*	pKS208::class 1 integron	Uncultured bacterium	AFS51540
37	YP_006957899	*aac(6′)-Ib-cr4*	pMdT	*SS. enterica* subsp. *enterica serovar* Typhimurium	YP_006957899,
					AFU63391
38	ADZ96942	*aac(6′)-Ib-cr*	Plasmid	*K. pneumoniae*	ADZ96942
39	CAA42873	*aac(6′)-Ib3, aac(6′)-Ib5*	plasmid pCFF04	*P. aeruginosa*	CAA42873
40	AAB24284	*aac(6′)-Ib4*	pSP21::class 1 integron,	*Se.* spp., uncultured bacterium, *En. cloacae*	AAB24284,
			pEl1573::class 1 integron		YP_006941442,
					YP_006965430
41	AAN41403	*aac(6′)-Ib11*	pSTI1::class 1 integron	*SS. enterica* subsp. *enterica* serovar Typhimurium	AAN41403
42	YP_006903338	*aac(6′)-Ib*	pNDM102337::class 1 integron,	*Escherichia coli*	YP_006903338,
			pNDM10505::class 1 integron		YP_006953195
43	YP_006959139	*aac(6′)-Ib*	pNDM10469::class 1 integron	*K. pneumoniae*	YP_006959139
44		*aac(6′)-Ib7*	Plasmid	*En. cloacae, Citrobacter freundii*	Not available
45		*aac(6′)-Ib8*	Plasmid	*En. cloacae*	Not available

The pJHCMW1-encoded AAC(6′)-Ib variant (accession number NP_608307) was subjected to BLASTP and the identical proteins were identified. Those that were closely related but not identical were identified by numbers. The names given in the publications or GenBank entries are shown. Those that were named aacA4 were named aac(6′)-Ib here.

**Table 2:** **Phenotypes of representative mutants of AAC(6′)-Ib**.

**Mutation**^**a**^	**AAC(6′)-Ib variant name**	**Phenotype**	**References**
Y80C		S	Panaite and Tolmasky, 1998
D117A		S	Pourreza et al., 2005
L119S	AAC(6′)-Ib′,	Specificity, Gm^r^ Ak^s^	Rather et al., 1992;
	AAC(6′)-Ib_7_,		Lambert et al., 1994;
	AAC(6′)-Ib^b^_8_		Casin et al., 2003
Q118L, L119S	AAC(6′)-Ib_11_	Specificity, Gm^r^ Ak^r^	Casin et al., 2003
L120A		S	Pourreza et al., 2005
Y166A		Specificity, Ak^s^ Km^r^	Shmara et al., 2001
E167A		S	Shmara et al., 2001
F171A		S	Shmara et al., 2001
F171L		Thermosensitive for Ak and Nm	Panaite and Tolmasky, 1998; Shmara et al., 2001
W104R, D181Y	AAC(6′)-Ib-cr	Expanded substrate spectrum including quinolones	Robicsek et al., 2006

S, susceptible.

a Numbering from sequence in accession number AF479774.

b The proteins differ at the amino terminus.

Subcellular localization studies of the AAC(6′)-Ib enzyme encoded by Tn*1331* showed that the enzyme is homogeneously distributed in the cytoplasmic compartment (Dery et al., 2003). AAC(6′)-Ib was one of three aminoglycoside modifying enzymes used in a study consisting of molecular dynamics simulations of the enzymes and aminoglycoside ribosomal RNA binding site, unliganded, and complexed with an aminoglycoside, kanamycin A. These studies concluded that the enzymes efficiently mimic the nucleic acid environment of the ribosomal RNA binding cleft (Romanowska et al., 2013). Extensive studies using mutagenesis showed some interesting phenotypes such as modifications in specificity, enhanced activity, or selective thermosensitivity (Table 2) (Panaite and Tolmasky, 1998; Chavideh et al., 1999; Shmara et al., 2001; Casin et al., 2003; Pourreza et al., 2005; Kim et al., 2007; Maurice et al., 2008). In addition, alanine scanning showed that several amino acid substitutions by A had minor effects. These mutagenesis studies together with structural and enzymatic analyses led to a deep understanding of features and characteristics of AAC(6′)-Ib proteins (Rather et al., 1992; Vetting et al., 2004; Maurice et al., 2008; Vetting et al., 2008; Ramirez and Tolmasky, 2010). The three dimensional structure of AAC(6′)-Ib and AAC(6′)-Ib_11_ have been experimentally determined in various conditions. AAC(6′)-Ib was crystallized in complex with coenzyme A and also in complex with both coenzyme A and kanamycin. The structures were solved to 1.8 Å and 2.4 Å resolution, respectively (Maurice et al., 2008). The broad spectrum variant AAC(6′)-Ib_11_ was crystallized in the absence of substrate and the structure was solved to 2.1 Å resolution (Maurice et al., 2008). These studies concluded that AAC(6′)-Ib exists as a monomer while AAC(6′)-Ib_11_ shows monomer/dimer equilibrium (Maurice et al., 2008). This was a somewhat surprising finding considering that previous studies had shown that two other acetyltransferases, AAC(6′)-Ii and AAC(6′)-Iy, exist as dimers (Wright and Ladak, 1997; Wybenga-Groot et al., 1999; Draker et al., 2003; Vetting et al., 2004; Wright and Berghuis, 2007; Vong et al., 2012). Interestingly, analysis of these crystal structures showed the presence of a flexible flap in AAC(6′)-Ib_11_ that may be the basis for its ability to utilize amikacin as well as gentamicin as substrates (Maurice et al., 2008). In another study a molecular model of AAC(6′)-Ib-cr has been generated (Maurice et al., 2008; Vetting et al., 2008), which led to postulate that the D181Y substitution is mainly responsible for modification in the strength of binding of the antibiotic substrate and that the substitution W104R stabilizes the positioning of Y181 (Robicsek et al., 2006; Strahilevitz et al., 2009).

**Figure 1:**
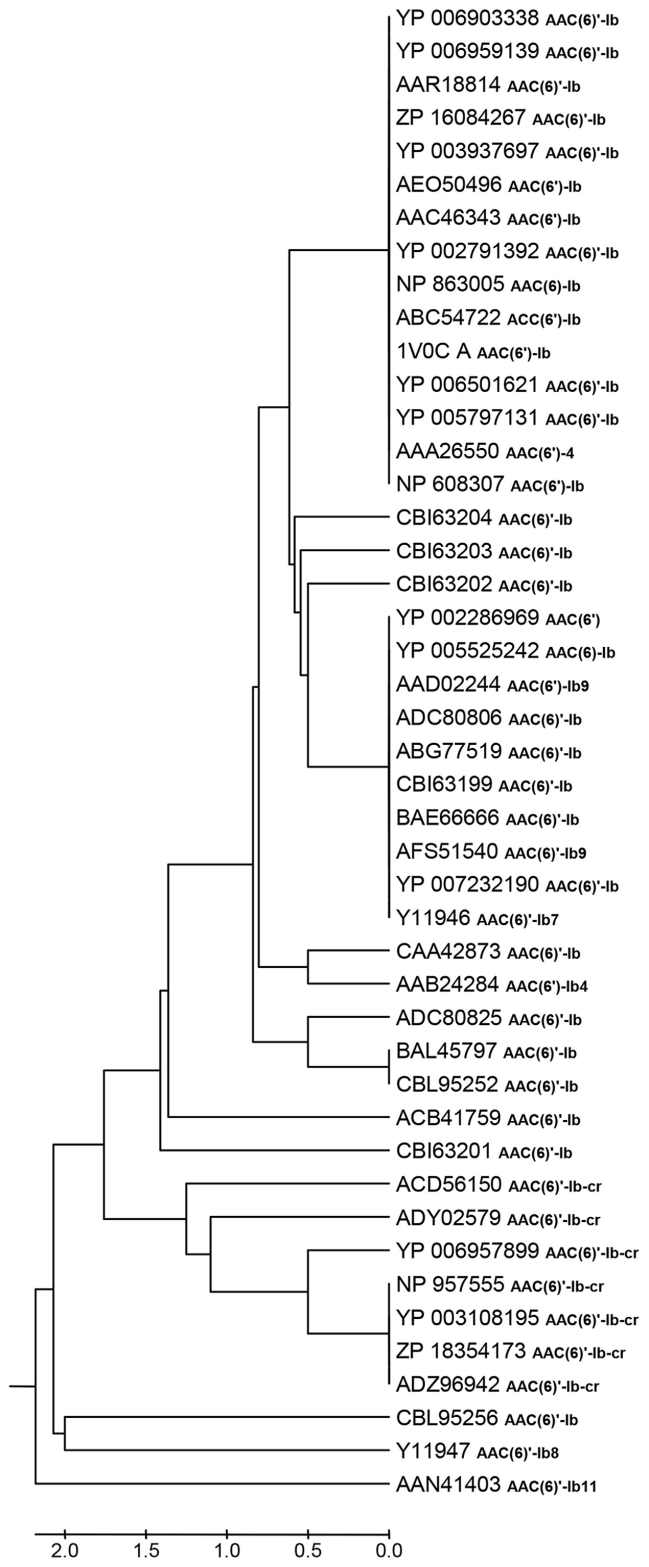
**UPGMA clustering analyses of 45 AAC(6′)-Ib protein sequences.** The optimal tree with the sum of branch length = 20.70628249 is shown. The evolutionary distances were computed using the number of differences method and are in the units of the number of amino acid differences per sequence. All positions containing gaps and missing data were eliminated. There were a total of 181 positions in the final dataset. Evolutionary analyses were conducted in MEGA5.

Table 1 shows that there are 45 non-identical AAC(6′)-Ib related entries in the NCBI database, 32 of which have identical name in spite of not having identical amino acid sequence. The N-termini of these proteins show the highest degree of heterogeneity with high variations in length stretching up to 60 amino acids, but these differences were suggested to be irrelevant (Casin et al., 1998; Maurice et al., 2008). Therefore, we defined a highly conserved central region composed of 181 amino acids shared by all proteins, which were compared using the MAFFT alignment algorithm (Katoh and Standley, 2013). Pairwise comparisons show that the sequences have 1 to 8 amino acid differences and a total of 24 positions showed amino acid variations. Moreover, clustering using the UPGMA algorithm (Sneath and Sokal, 1973) defined 18 sequence clusters, 14 of which consist of a singleton, and 4 of which include 2–16 proteins (Figure 1). Different clusters can exhibit similar properties while others show substantial differences in their characteristics such as those cases in which there are significant specificity variations like extended substrate spectrum as described in the above paragraphs.

## The *aac(6′)-Ib* gene

**Figure 2:**
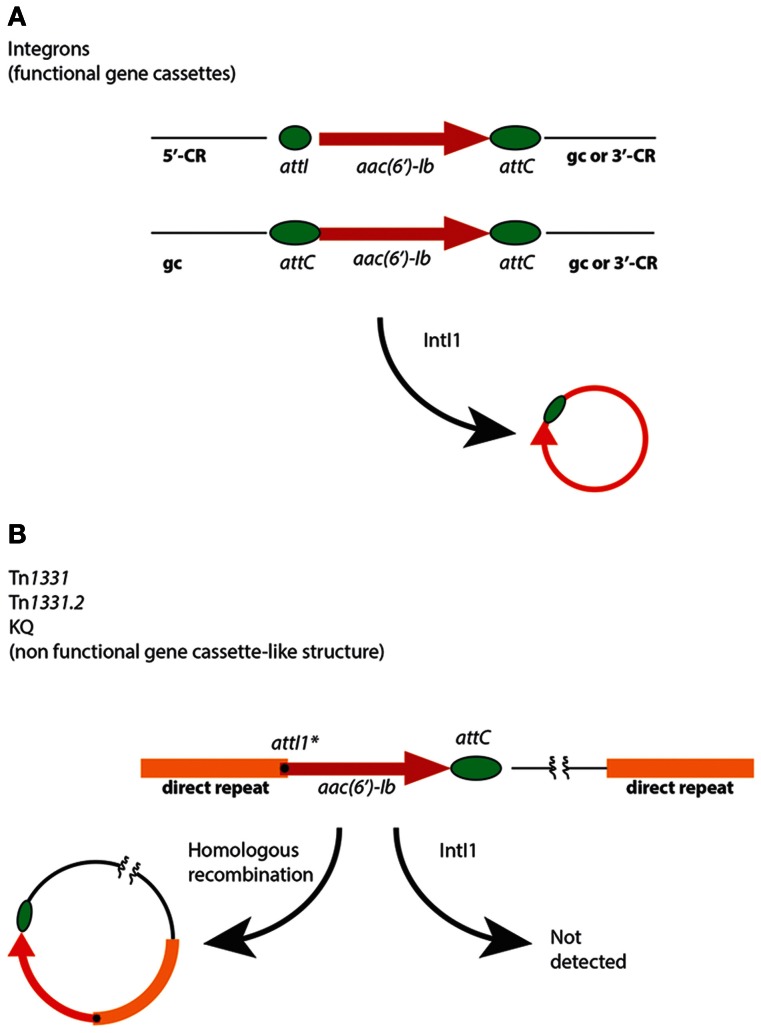
**Mobilization of *aac(6′)-Ib*. (A)** Generic genetic maps of integrons in which an *aac(6′)-Ib* gene cassette is located immediately following the 5′ conserved region (5′-CR) (top map) or following one or more gene cassettes (gc) inside the variable portion, and followed by other gene cassettes or the 3′ conserved region (3′-CR) (bottom map). The small green ellipse represents *attI* and the big green ellipses represent *attC*. **(B)** Relevant portion of the Tn*1331*, Tn*1331.2*, and KQ elements (Tolmasky and Crosa, 1987; Tolmasky et al., 1988; Sarno et al., 2002; Rice et al., 2008). For clarity Tn*1332*, which has a more complicated structure in its direct repeats (Poirel et al., 2006), is not shown, but it could experience mobilization by homologous recombination as shown. The black dot represents attI1^*^. The homologous recombination pathway for generation of an *aac(6′)-Ib*-containing circular molecule has been proposed by Zong et al. (2009).

The *aac(6′)-Ib* genes are usually found as fully functional or deficient gene cassettes associated to class 1 integrons, insertion sequences such as IS*26*, and truncated or disrupted integrons (Figure 2 and Table 1) (Sarno et al., 2002; Woodford et al., 2009; Ramirez and Tolmasky, 2010). These genetic elements may be part of plasmids, transposons, genomic islands, or other structures such as the KQ element (Rice et al., 2008), which together contribute to the gene's ability to disseminate at the cellular and molecular levels (Tolmasky, 2007b). When present in integrons, *aac(6′)-Ib* gene cassettes can be found located adjacent to the 5′-conserved region, i.e., flanked by *attI* and *attC*, or internal to the variable portion containing *attC* loci at both ends (Figure 2). In both cases, as expected, the gene cassette can be mobilized by the integrase IntI1 (Figure 2) (Cambray et al., 2010; Hall, 2012). In addition, a gene cassette-like structure containing *aac(6′)-Ib*, composed of a copy of attI1^*^ at the beginning of the structural gene and a regular *attC* downstream of it (see Figure 2), was found as part of a region resembling the variable portion of integrons in Tn*1331* (Woloj et al., 1986; Tolmasky et al., 1988; Tolmasky, 1990; Tolmasky and Crosa, 1993; Sarno et al., 2002), Tn*1331.2* (Tolmasky and Crosa, 1991), Tn*1332* (Poirel et al., 2006), the KQ element (Rice et al., 2008), a Tn*1331* derivative recently isolated from a clinical *Klebsiella pneumoniae* strain belonging to the ST512, which derived from the ST258, known to be spread worldwide (Chen et al., 2012; Garcia-Fernandez et al., 2012; Warburg et al., 2012), and a complex mosaic region present in the chromosome of *Proteus mirabilis* JIE273 (Zong et al., 2009). Assays overexpressing IntI1 in cells containing Tn*1331* were unable to detect any excision of the *aac(6′)-Ib* gene cassette-like structure, suggesting that it is nonfunctional or it excises at an extremely low efficiency (Ramirez et al., 2008). Interestingly, despite the IntI1-mediated lack of mobility of this DNA region, the gene could be mobilized by means of a mechanism recently proposed by Zong et al. that occurs through homologous recombination between 520-bp direct repeats located upstream and downstream of the gene cassette-like structure (Figure 2) (Zong et al., 2009).

These multiple locations taken together with the ability of the genetic elements to spread at the molecular and cellular level provide *aac(6′)-Ib* genes with the capability to reach virtually all gram-negatives and other undetermined bacteria such as those that are still unculturable. The gene has also been found in plasmids harboring resistance genes of high importance such as the recently described *ndm-1* (Yong et al., 2009; Bonnin et al., 2012; Villa et al., 2012).

## Inhibition

The rise in drug resistance affects all known antibiotics and has been identified as one of the greatest threats to human health. Therefore, there is an immediate need for new agents with activity against multiresistant bacteria and at the present moment there is no evidence that this need will be fully met in the near future (Boucher et al., 2009). A group of pathogens that cause the majority of hospital infections, named ESKAPE (*Enterococcus faecium, Staphylococcus aureus, K. pneumoniae, Acinetobacter baumannii, P aeruginosa*, and *Enterobacter*), is becoming highly resistant to antibiotics including aminoglycosides (Rice, 2008). They carry aminoglycoside modifying enzymes genes, and one of the most common in the gram-negative members is *aac(6′)-Ib* (Ramirez and Tolmasky, 2010; Shaul et al., 2011; Herzog et al., 2012). An obvious solution to this problem would be the development of new aminoglycosides, a strategy that is being pursued using numerous approaches (Green et al., 2010, 2011; Houghton et al., 2010). A variety of new aminoglycoside derivatives including chemical modification of existing aminoglycosides, aminoglycoside dimers, or aminoglycoside-small molecule conjugated are being produced and tested (reviewed in Houghton et al., 2010). In particular plazomicin (ACHN-490), a novel neoglycoside derived from sisomicin that carries a hydroxymethyl group at position 6′, has shown enhanced activity against multiresistance gram-negatives and gram-positives including strains carrying *aac(6′)-Ib* (Endimiani et al., 2009; Landman et al., 2011).

Others are approaching the problem in such a way that the existing aminoglycosides continue to be effective by designing enzymatic inhibitors that can act in combination with the antibiotic, mimicking the strategy successfully used to curb resistance to β-lactams (Williams and Northrop, 1979; Daigle et al., 1997; Haddad et al., 1999; Liu et al., 2000; Burk and Berghuis, 2002; Boehr et al., 2003; Draker et al., 2003; Gao et al., 2005, 2006; Welch et al., 2005; Lombes et al., 2008; Magalhaes et al., 2008; De Pascale and Wright, 2010; Drawz and Bonomo, 2010; Green et al., 2012; Vong et al., 2012). However, these efforts are still scarce when one compares them to those invested to discover and design β-lactamase inhibitors. Furthermore, the attempts to find inhibitors of AAC(6′)-Ib have only yielded a compound, synthesized using non-aminoglycoside-like fragments, with a rather modest level of inhibition of AAC(6′)-Ib (Lombes et al., 2008).

An alternative approach that is being explored is silencing expression of the resistance gene. Early attempts at interfering with expression of *aac(6′)-Ib* consisted of identifying regions available for interaction with antisense oligonucleotides in a monocistronic *in vitro* synthesized mRNA by RNase H mapping in combination with computer prediction of its secondary structure (Sarno et al., 2003). The selected sites were used as targets for a collection of oligodeoxynucleotides, of which some had the ability to induce RNase H-mediated *in vitro* degradation of the mRNA, inhibited *in vitro* synthesis of the enzyme in coupled transcription/translation assays, and upon delivery by electroporation significantly reduced the number of cells surviving after exposure to amikacin (Sarno et al., 2003). The mechanism of this *in vivo* inhibition is most probably through RNase H digestion of the mRNA, but other possibilities such as steric hindrance cannot be discarded at this time. Alternatively, modest but significant inhibition of expression of *aac(6′)-Ib* was achieved by applying EGS technology, in which short antisense RNA molecules, known as external guide sequences, are used to elicit RNase P-mediated degradation of a target mRNA (Guerrier-Takada et al., 1997; Lundblad and Altman, 2010). Initially, *E. coli* harboring *aac(6′)-Ib* were transformed with recombinant clones specifying the appropriate RNA oligonucleotide sequences under an inducible promoter. The transformed derivatives were then cultured in the presence of amikacin under conditions of expression of the external guide sequences. The results showed that in a few cases the external guide sequences induced a reduction of the minimal inhibitory concentration of amikacin (Soler Bistue et al., 2007). These results were considered proof of concept, but the strategy was not viable because antisense oligonucleotides must be added from the milieu and find their way inside the cells without being degraded. Thus, nuclease resistant oligonucleotide analogs that still induce inhibition of gene expression by RNase P activation had to be found. Out of a variety of oligoribonucleotide analogs including 2′-*O*-methyl oligoribonucleotides, phosphorodiamidate morpholino oligomers, phosphorothioate oligodeoxynucleotides, or locked nucleic acids (LNA)/DNA co-oligomers that were tested, LNA/DNA co-oligomers with certain configurations were found to be capable of eliciting RNase P-mediated cleavage of mRNA *in vitro* (Soler Bistue et al., 2009). Following this finding, a selected LNA/DNA co-oligomer was added to the hyperpermeable *E. coli* AS19 harboring *aac(6′)-Ib* and it was found that growth was inhibited in the presence of amikacin, indicating that the compound may have induced RNase P-mediated inhibition of expression of the gene (Soler Bistue et al., 2009). These results were encouraging but it must be noted that inhibition of expression of *aac(6′)-Ib* is still far from being a viable option to overcome aminoglycoside resistance in the clinical setting. Several problems remain to be solved like inducing penetration of the oligonucleotide analogs inside wild type cells in enough quantities to exert the biological activity or achieve enough inhibition levels in spite of the usual presence of multiple copies of the gene due to its inclusion in high copy number plasmids. Toward finding solutions to these problems, recent experiments suggest that LNA/DNA co-oligomers may be able to reach the cytoplasm of untreated cells at low efficiency (Traglia et al., 2012). Strategies will have to be developed to increase the efficiency of delivery inside bacterial cells.

### Conflict of interest statement

The authors declare that the research was conducted in the absence of any commercial or financial relationships that could be construed as a potential conflict of interest.
